# Cyanide is an endogenous stimulator of endothelial cell proliferation, migration and differentiation

**DOI:** 10.3389/ebm.2026.10856

**Published:** 2026-01-23

**Authors:** Anna Kieronska-Rudek, Maria Petrosino, Karim Zuhra, Csaba Szabo

**Affiliations:** Section of Pharmacology, Department of Oncology, Microbiology and Immunology, Faculty of Science and Medicine, University of Fribourg, Fribourg, Switzerland

**Keywords:** vascular, endothelial, proliferation, migration, blood vessels

## Abstract

Cyanide is generally considered a cytotoxic molecule. However, recent studies have shown that mammalian cells — including endothelial cells — can produce cyanide from glycine via a lysosomal pathway. Studies in hepatocytes indicated that cyanide, when administered at low concentrations, or when generated from endogenous sources, exerts regulatory, rather than cytotoxic effects. Here we show that human umbilical vein endothelial cells produce detectable levels of cyanide (∼0.1 nmoles/mg protein/h), and this is enhanced by administration of glycine (1 mM). Glycine stimulates endothelial cell proliferation, migration and tube formation. Low concentrations of the cyanide releasing molecules amygdalin or mandelonitrile (100 µM) exert similar effects. On one hand, cyanide induces the upregulation of VEGF protein in endothelial cells, while on the other hand, VEGF stimulates the generation of cyanide by endothelial cells, suggesting a positive feedback. VEGF-stimulated endothelial cell ATP generation, proliferation and migration is inhibited by the cyanide scavenger hydroxycobalamin (10 µM) as well as by pharmacological agents that prevent lysosomal acidification and thus inhibit cyanide formation by the endothelial cells. In conclusion, cyanide, at low concentrations, generated by endothelial cells, acts as a proangiogenic mediator, via stimulation of the VEGF pathway and the maintenance of cellular bioenergetics.

## Impact statement

This study identifies cyanide as an endogenous signaling molecule that promotes endothelial cell proliferation, migration, and differentiation. By demonstrating a physiological role for low-level cyanide generation in vascular biology, the work challenges the traditional view of cyanide solely as a toxicant and reveals a previously unrecognized regulatory pathway with broad implications for endothelial function and angiogenesis.

## Introduction

Cyanide is primarily recognized as a cytotoxic agent, which inhibits mitochondrial electron transport through binding to the heme a3 group in mitochondrial Complex IV (Cytochrome C oxidase, CCOx) [1]. However, a separate body of evidence suggests that cyanide, at low concentrations, can also exert various cytoprotective or regulatory actions [2–4]. Most recently, an endogenous cyanide generating pathway has been demonstrated in mammalian cells and tissues. According to this pathway, cyanide is produced from glycine within lysosomes under acidic conditions and cyanide biogenesis supports cellular bioenergetics and proliferation and exerts cytoprotective effects in hepatocytes [4]. Although this recent report was primarily focusing on hepatocytes, a survey of various cell lines and primary cells also demonstrated that endothelial cells also produce detectable levels of cyanide, and this cyanide generation can be stimulated by increasing the extracellular glycine concentration [4]. However, the functional role of cyanide generation by endothelial cells has not yet been elucidated; the available body of literature on the effects of cyanide in this cell type focuses on the toxicological context [5].

Here we demonstrate that cyanide generation in endothelial cells, at low concentrations, stimulates cell proliferation and angiogenesis. The current findings put cyanide in a physiological, regulatory context within the cardiovascular system.

## Materials and Methods

### Materials

Mandelonitrile (#116025), amygdalin (#A6005), glycine (#G7126), hydroxychloroquine (#H0915), Vitamin B12a (#H1428000), vascular endothelial growth factor (VEGF, #SRP3182), Corning® Matrigel® Basement Membrane Matrix (#CLS354234) and β-actin antibody (#A1978) were purchased from Sigma-Aldrich (Buchs, Switzerland). LysoTracker™ Bafilomycin A1 (J61835) was purchased from Alfa Aesar/ThermoFisher (Haverhill, MA, United States). Deep Red (#L12492) was purchased from ThermoFisher Scientific/Invitrogen. The anti-VEGF antibody (#ab51745) was purchased from Abcam (Cambridge, UK). The cyanide detection probe Chemosensor P (2-amino-3-((5-(benzothiazol-2-yl)-2-hydroxybenzylidene)amino) maleonitrile) [6] was a kind gift of Dr. Sait Malkondu (Giresun University, Giresun, Turkey).

### Cell culture

Human umbilical vein endothelial cells (HUVECs, CC-2519) were purchased from Lonza (Basel, Switzerland). Cells were maintained in EGM®-2 Endothelial Cell Growth Medium-2 BulletKit® (CC-3162; Lonza, Basel, Switzerland) at 37 °C in a humidified atmosphere containing 5% CO_2_. Cells up to the 5th passage were used.

### Electrochemical analysis of cyanide production

Electrochemical detection of cyanide was performed following a previously described protocol [4], with minor modifications. Briefly, cells were seeded in 24-well plates and allowed to adhere overnight. Depending on the experimental conditions, cells were then incubated for 72 h in medium supplemented with VEGF (20 ng/mL) or control medium without VEGF.

To assess cyanide generation, cells were treated for 16 h with the cyanide-releasing compound: glycine (1 mM), amygdalin (100 μM), or mandelonitrile (100 μM) or control vehicle. In another experiment, cells were incubated in the absence or presence of VEGF for 72 h (see below). Following the various treatments, culture media were collected and mixed immediately in a 1:1 ratio with 1 M NaOH to stabilize released hydrogen cyanide (HCN) as CN^−^. The samples were incubated at room temperature for 30 min to ensure complete conversion of HCN to CN^−^. A standard curve was generated using potassium cyanide (KCN). Cyanide production rates were normalized to total protein content, which was determined using the BCA protein assay (Thermo Scientific, #23225). Results were expressed as nanomoles of cyanide per milligram of protein per hour. The specificity of this method has previously been validated [4].

### Western blotting

Cell lysates were prepared using RIPA Lysis and Extraction Buffer (Thermo Fisher Scientific), supplemented with Halt™ Protease and Phosphatase Inhibitor Cocktail (Thermo Fisher Scientific). Total protein concentrations were determined using the Pierce™ BCA Protein Assay Kit (Thermo Fisher Scientific). Western blot analysis was performed according to a previously described protocol [7]. VEGF protein expression was detected using a primary antibody against VEGF (1:1,000), followed by incubation with a horseradish peroxidase-conjugated secondary antibody (Cell Signalling Technology, Danvers, MA, United States). After VEGF detection, membranes were stripped using Restore™ PLUS Western Blot Stripping Buffer (#10016433, Thermo Fisher Scientific) and incubated with primary antibody against β-actin (1:15,000) as a loading control. Blots were developed using Radiance Plus Chemiluminescence Substrate (#AC2103, Azure Biosystems, Dublin, CA, United States). Densitometric analysis was performed using the ImageJ software (NIH, Bethesda, MD, United States). Protein levels were normalized to β-actin.

### Confocal microscopy-based detection of cyanide

Cells were seeded in 4-chamber glass-bottom dishes (pre-coated with rat tail collagen, #354236, Corning) at a density of 2.5 × 10^4^ cells per compartment. Cells were incubated overnight at 37 °C in a humidified incubator (5% CO_2_) to allow attachment. Cells were then incubated in EGM-2 media with or without VEGF for 72 h.

Cyanide levels were assessed using a previously established protocol [4, 8] with slight modifications. Briefly, cells were incubated for 1 h with 10 μM of the cyanide-selective fluorescent probe CSP. After probe incubation, cells were stained with LysoTracker™ Deep Red (50 nM) for 10 min to label lysosomes. Cells were then washed 3x with Hank’s Balanced Salt Solution (Thermo Fisher Scientific) to remove excess dye. Confocal imaging was performed using a Leica SP5 microscope equipped with a ×40 oil-immersion objective. For each condition, three images per well were acquired from five independent biological replicates. Fluorescence intensity was quantified using the ImageJ software. The specificity of this method has previously been validated [4].

### Cell proliferation assay

Cells were seeded in 96-well plates at a density of 4 × 10^3^ cells per well and incubated overnight at 37 °C in a humidified incubator to allow cell adhesion. The following day, the culture medium was replaced with medium containing various pharmacological agents including glycine (1 mM), or amygdalin (100 μM), or mandelonitrile (100 μM). Immediately after treatment, the plate was placed in the BioTek Cytation 5 Cell Imaging Multimode Reader (Agilent) [4]. Live-cell imaging was performed every 4 h over 48 h, with four images captured per well at ×100 magnification.

In another set of experiments, cells were placed either in a modified EGM-2 BulletKit® (Lonza) medium which excluded all growth factors and contained a reduced amount of FBS (0.5% FBS) with or without VEGF (20 ng/mL) supplementation in the presence or absence of hydroxocobalamin (B12a, 10 μM), hydroxychloroquine (HCQ, 10 μM) or bafilomycin (30 nM) and cell proliferation was monitored for 48 h as described above.

### Wound healing assay

Cells were seeded in 96-well plates at a density of 2.5 × 10^4^ cells per well and incubated overnight to allow for cell adherence. The following day, scratch wounds were generated using the Essen BioScience WoundMaker (4563; Essen BioScience Inc., Hertfordshire, UK). Cells were then gently washed with PBS to remove debris resulting from the scratch. The medium was subsequently replaced with treatment medium containing either glycine (1 mM), or amygdalin (100 µM) or mandelonitrile (100 µM). The plate was then placed in a BioTek Cytation 5 Cell Imaging Multimode Reader, and images were captured every 4 h over 20 h. Four images per well were acquired at ×100 magnification.

### Tube formation assay

Prior to cell seeding, a 96-well plate was coated with 60 μL of Matrigel per well and incubated at 37 °C for 1 h to allow the matrix to polymerize. Cells were then seeded at a density of 1.5 × 10^4^ cells per well in 100 μL of EBM-2. The plate was returned to the incubator for 2 h to allow for cell attachment, minimizing potential interference from treatment compounds during the adhesion phase. Following this initial incubation, test compounds were added to the appropriate wells to achieve final concentrations of 1 mM glycine and 100 μM amygdalin or mandelonitrile. Incubation continued for an additional 14–16 h at 37 °C when analysis was conducted as described [9]. Four representative images per well were captured using a BioTek Cytation 5 Cell Imaging Multi-Mode Reader at ×100 magnification. Image analysis was performed using the Angiogenesis Analyzer plugin for ImageJ. Quantitative assessment of angiogenesis was based on key morphological parameters, including total tube length, number of segments, and number of junctions.

### Adenosine triphosphate (ATP) production

ATP levels were measured using the CellTiter-Glo® Luminescent Cell Viability Assay (Promega, Madison, WI, United States) as described [10]. Luminescence was measured using a Infinite 200 Pro plate reader (Tecan).

### Statistical analysis

Unless otherwise stated, data are presented as mean values ± SEM of several independent experiments where an independent experiment is defined as an experiment performed on a different experimental day, representing a biological replicate (as opposed to technical replicates). No statistical methods were used to pre-determine sample sizes, but our sample sizes are similar to those reported in previous publications [4, 8]. Data are presented as mean ± SEM of 4-5 biological experiments, where the average of a minimum of three technical replicates is calculated and entered as a single value. Paired or unpaired Student's t-test was used to compare 2 groups, as appropriate. For multiple comparisons, one or two-way ANOVA followed by post-hoc analysis by Dunnett or Fisher LSD test, respectively. Differences among groups are considered statistically significant for p values of ≤0.05. Statistical analysis was performed using GraphPad Prism 10.2 (GraphPad Software Inc., San Diego, California, United States).

## Results

**FIGURE 1:**
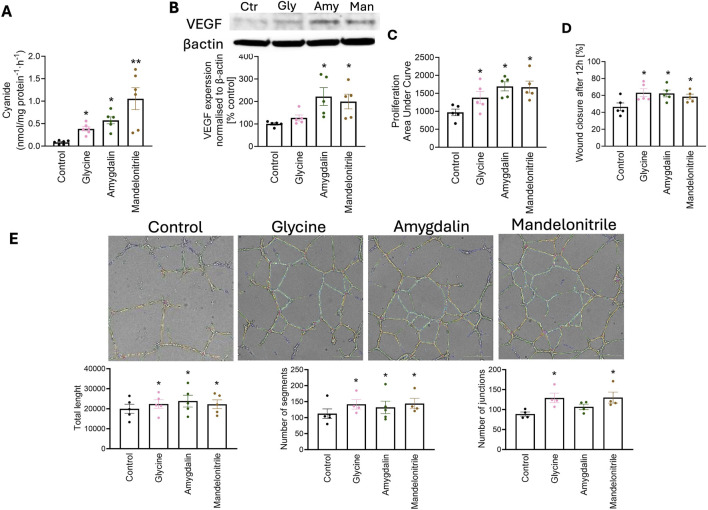
Low levels of cyanide promote endothelial cell proliferation, migration and differentiation. **(A)** Detection of cyanide generation **(B)**, induction of VEGF expression **(C)**, proliferation **(D)**, wound healing and **(E)** tube formation in HUVECs in response to incubation with glycine (Gly) (1 mM), amygdalin (Amy) (100 μM) or mandelonitrile (Man) (100 μM). For **(E)**, representative images as well as statistical analysis of the data are shown. Data are presented as mean ± SEM. Individual data points represent independent biological replicates. Statistical analysis was performed as described in the Materials and Methods section; significance is indicated as follows: *p < 0.05, **p < 0.01.

HUVECs grown in culture in Vascular Cell Basal Medium (which contains 30 µM glycine) produce detectable levels of cyanide (0.1 nmoles/mg protein/h); cyanide generation was enhanced by approximately 4-fold when the medium was supplemented with glycine (1 mM) to 0.4 nmoles/mg protein/h (Figure 1A). Glycine induced a trend toward increased expression of vascular endothelial growth factor (VEGF) (Figure 1B) and stimulated cell proliferation (Figure 1C), migration (Figure 1D) and tube formation (Figure 1E). A stimulatory effect on VEGF expression was also observed after treatment of the cells with low concentrations of the cyanogenic molecules amygdalin (100 μM) or mandelonitrile (100 μM) (Figure 1B); both of these compounds–which release cyanide at a comparable rate as the endogenous cyanide generation rates [4] – mimicked the effect of glycine and stimulated endothelial cell proliferation, migration and tube formation (Figures 1C-E). These compounds, when added to HUVECs, generate cyanide at rates of 0.5–1 nmoles/mg protein/h (Figure 1A).

**FIGURE 2:**
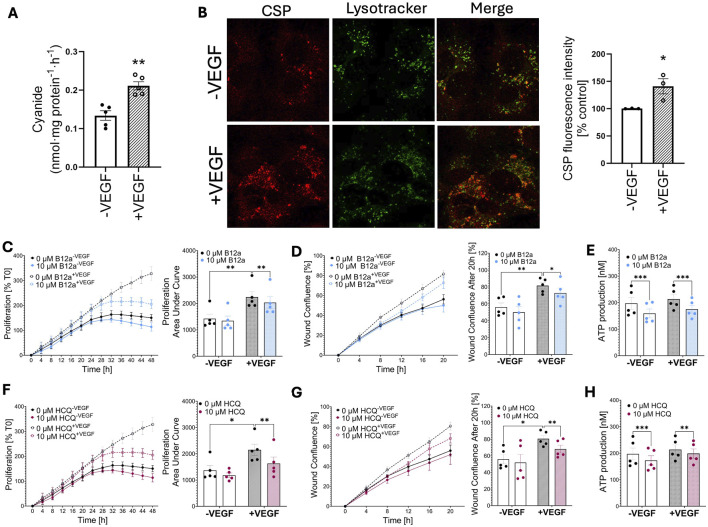
Endogenous cyanide generation supports endothelial cell proliferation, migration and differentiation. **(A)** Detection of cyanide generation by the electrochemical method and **(B)** confocal microscopy using the cyanide-specific probe CSP in HUVECs incubated with or without VEGF for 72 h. Effect of cyanide scavenger Vitamin B12a (B12a) **(C–E)** or hydroxychloroquine (HCQ) **(F–H)** in HUVECs in the presence of absence of 20 ng/mL VEGF on **(C,F)** cell proliferation, **(D,G)** wound healing, and **(E,H)** ATP production. Data are presented as mean ± SEM. Individual data points represent independent biological replicates. Statistical analysis was performed as described in the Materials and Methods section; significance is indicated as follows: *p < 0.05, **p < 0.01, ***p < 0.001.

Administration of VEGF stimulated the generation of cyanide by endothelial cells (Figures 2A,B); confocal imaging studies localized cyanide primarily to the lysosomes (Figure 2B) — consistently with the prior findings in hepatocytes and fibroblasts [4, 8]. A less pronounced cyanide signal was also detected in the cytosolic compartment of the cells (Figure 2B).

Scavenging of cyanide with hydroxocobalamin (Vitamin B12a, 10 µM) reduced the VEGF-induced cell proliferation (Figure 2C), migration (Figure 2D) and ATP production (Figure 2E). The lysosomal deacidification agent hydroxychloroquine, which inhibits cyanide generation by shifting the pH optimum of the reaction in the lysosomes [4], also inhibited VEGF-stimulated proliferation (Figure 2F), migration (Figure 2G) and ATP production (Figure 2H); similar inhibitory effect was also detected by treatment of the cells with the lysosomal proton pump inhibitor bafilomycin, which attenuated basal and VEGF-stimulated proliferation by 21 ± 4 and 45 ± 3%, respectively (n = 4, <0.05).

## Discussion

With recent data demonstrating its lysosomal generation from glycine in mammalian cells [4], cyanide can be classified as a gasotransmitter: a diffusible, endogenous mediator that is produced in mammalian cells to serve regulatory functions [4, 11]. Importantly, several gasotransmitters have previously been shown to be produced in endothelial cells. NO, produced from L-arginine, is responsible for endothelium-dependent vascular relaxations, and serves as a pro-angiogenic agent [12–16]. CO from heme oxygenase serving as an endothelial cell protective mediator [17–19], and H_2_S exerts vasorelaxant and proangiogenic effects [20–23]. The current report adds cyanide as a pro-angiogenic molecule. Interestingly, prior reports have made important connections of VEGF to the NO and H_2_S systems. VEGF has been shown to induce both the generation of NO and H_2_S in endothelial cells, and these two mediators [24, 25] — in a synergistic manner [26] — are important effectors of VEGF’s proangiogenic effect. In addition, H_2_S has also been shown to upregulate VEGF and VEGF receptor expression and activation [27–30].

The current findings indicate that cyanide, when produced at low levels by endothelial cells, exerts similar regulatory actions: it supports cell proliferation, migration and differentiation. The effects of cyanide biogenesis could be mimicked by exposure of the cells to low levels of the cyanogenic compounds amygdalin or mandelonitrile, which generated comparable, or slightly higher levels of cyanide, than the cyanide generation rate observed in response to addition of glycine. (The concentration of glycine, used at 1 mM in this study, is slightly above its physiological plasma levels, which are in the 0.2–0.5 µM range).

The current report demonstrates that Vitamin B12a (a potent scavenger of cyanide [31]), as well as hydroxychloroquine and bafilomycin (compounds that inhibits cyanide generation via inhibiting lysosomal acidification) reduce the VEGF-induced proliferative response in HUVECs. Although all of these pharmacological agents have several different pharmacological actions — for instance, Vitamin B12 can scavenge H_2_S and other reactive species too [31] — the above findings, coupled with the effect of two structurally different cyanide-releasing molecules, which mimic the effect of glycine, point to the role of the endogenous cyanide pathway in the observed actions. Moreover, the upregulation of VEGF in response to cyanogenic compounds suggests a potential positive feedback cycle between VEGF and cyanide.

Confocal imaging of HUVECs produced data that are consistent with the lysosomal cyanide generation pathway already demonstrated in various cell types, including hepatocytes, fibroblasts and various cancer cells [4]. According to this pathway, glycine is taken up into the lysosomes, and reacts with HOCl to generate various short-lived intermediary species, which, in turn, decompose to yield cyanide and CO_2_. Indeed, prior studies have already demonstrated all of the relevant [4] components of the glycine/cyanide biogenetic system in endothelial cells, including peroxidase activity, HOCl generation, uptake of glycine from the extracellular space as well as the serine/glycine conversion system governed by the intracellular enzyme serine hydroxymethyl transferase [32–35].

Importantly, prior studies have already demonstrated that glycine can stimulate endothelial cell proliferation and angiogenesis, and attributed its effects to a bioenergetic stimulatory response [36]. Likewise, at relatively low concentrations (1–10 mM), glycine was found to stimulate angiogenesis and vascular development in zebrafish embryos [37]. There are also several lines of data demonstrating cytoprotective effects of glycine in endothelial cells [38]. The current findings are consistent with the above findings. We suggest that — similar to what was demonstrated in hepatocytes and hepatoma cells — also in HUVECs, low concentrations of endogenously generated cyanide stimulate mitochondrial electron transport and ATP generation, which supports the energy requirement of proliferation, migration and differentiation.

Cyanide is known as a potent inhibitor of Complex IV (cytochrome c oxidase), and this effect is primarily attributed to its cytotoxic actions. This effect, however, is expected to be less relevant in endothelial cells for two reasons: (a) the levels of cyanide generated are rather low, and we have found no evidence of cytotoxic or cytostatic effects, and, in fact, a stimulatory effect on bioenergetics was noted; (b) endothelial cells tend to utilize glycolysis, rather than oxidative phosphorylation to meet their energetic demands [39]. It is interesting to notice that in the zebrafish study mentioned earlier, at higher concentrations (100–400 mM), the stimulatory effect of glycine was diminished and the amino acid inhibited vascular development [37]. Based on prior data — in cells overproducing cyanide due to silencing of the cyanide degradation pathway governed by rhodanese [4], or cells that exhibit genetic mutations in the glycine degradation pathway [4], or cells that contain high levels of cyanide due to inhibition of rhodanese [11] — such a biphasic concentration-response is consistent with the action of cyanide as an endogenous mediator.

The data presented in this article indicate that cyanide production exerts regulatory roles in endothelial cells, and promotes endothelial cell proliferation, migration and differentiation. Although the underlying effects remain to be further characterized, the data presented in this report point, in part, to a potential VEGF-related mechanism, and in part to stimulation of cellular bioenergetics. A limitation of the current study is that cellular bioenergetic parameters (e.g., oxidative phosphorylation, glycolysis) have not been measured; such measurements would further expand on the underlying mechanisms of cyanide’s action. Given the fact that cyanide is an endogenous molecule, which is present in the blood at detectable levels (basally, in approximately 300–600 nM) [1, 4], the current report raises the possibility that low levels of cyanide may contribute to the maintenance of endothelial and cardiovascular homeostasis. It is noteworthy in this respect that there are several reports showing that the cyanide metabolite 2-aminothiazoline-4-carboxylic acid correlates with various clinical parameters in cardiovascular disease and aging [40, 41].

## Data Availability

The original contributions presented in the study are publicly available. The data can be found here: https://doi.org/10.5281/zenodo.18154411.
